# Low-density lipoprotein cholesterol modulates amyloid-related entorhinal vulnerability and episodic memory performance in older adults

**DOI:** 10.3389/fnagi.2026.1933036

**Published:** 2026-09-24

**Authors:** Minh Le Tran, Kahye Kim, Jaeuk U. Kim, Yu Yong Choi, JunHyeong Do, Kun Ho Lee, Ji-Woo Seok

**Affiliations:** 1 Division of Digital Health Research, Korea Institute of Oriental Medicine, Daejeon, Republic of Korea; 2 Korean Convergence Medical Science, University of Science and Technology, Daejeon, Republic of Korea; 3 Gwangju Alzheimer’s & Related Dementias (GARD) Cohort Research Center, Chosun University, Gwangju, Republic of Korea; 4 Department of Biomedical Science, Chosun University, Gwangju, Republic of Korea; 5 Dementia Research Group, Korea Brain Research Institute, Daegu, Republic of Korea

**Keywords:** Alzheimer’s disease, amyloid-beta, entorhinal cortex, episodic memory, low-density lipoprotein cholesterol, moderated mediation

## Abstract

**Introduction:**

Cerebral amyloid-beta (Aβ) burden does not fully explain variation in Alzheimer’s disease (AD)-related brain structure and episodic memory. We examined whether circulating low-density lipoprotein cholesterol (LDL-C) modifies the cross-sectional statistical association between Aβ burden, the entorhinal cortex (EC) thickness and episodic memory performance.

**Methods:**

Cross-sectional data from 1,253 individuals in the Gwangju Alzheimer’s Disease and Related Dementias cohort (798 cognitively normal, 425 mild cognitive impairment, 30 AD dementia) were analyzed. Primary conditional-process models were adjusted for age, sex, education, body mass index, and HbA1c; clinical diagnosis was examined in sensitivity analyses. HC3 heteroskedasticity-consistent inference was used for the focal interaction, and indirect associations were estimated using 10,000 bootstrap resamples.

**Results:**

Greater Aβ burden was associated with lower EC thickness (β = −0.0863, *p* = 0.0012), and EC thickness was positively associated with episodic memory (β = 0.3809, *p* < 0.001). The unconditional indirect statistical association was supported (β = −0.0329, 95% bias-corrected bootstrap CI [−0.0647, −0.0072]). LDL-C modified the Aβ–EC association (β = −0.0937, HC3 SE = 0.0332, 95% CI [−0.1589, −0.0285], *p* = 0.0049), although the incremental explanatory magnitude was small (ΔR^2^ = 0.0082). The conditional Aβ–EC association was unsupported at approximately 66 mg/dL LDL-C (β = 0.0263, *p* = 0.583), not statistically supported at approximately 100 mg/dL (β = −0.0639, *p* = 0.060), and negative at 138 mg/dL (β = −0.1648, *p* < 0.001). The index of moderated indirect association was supported (β = −0.0357, 95% bias-corrected bootstrap CI [−0.0637, −0.0119]); conditional indirect associations were most clearly supported at higher LDL-C levels. A similar interaction was observed for non-HDL cholesterol, suggesting that the pattern may reflect a broader atherogenic lipid context.

**Conclusion:**

Circulating LDL-C was associated with modest heterogeneity in the cross-sectional Aβ–EC relationship, with more clearly negative conditional associations observed at higher LDL-C levels. Longitudinal studies are needed to determine the temporal and clinical relevance of these findings.

## 1 Introduction

Alzheimer’s disease (AD) is now increasingly conceptualized as a multiyear biological process, with brain amyloid-beta (Aβ) accumulation as an early and characteristic pathology (Musiek and Holtzman, 2012). However, Aβ load alone is insufficient to account for the timing, location and extent of subsequent neurodegeneration and cognitive impairment (Knopman et al., 2019). Individuals with comparable levels of cerebral Aβ may show substantially different degrees of structural and cognitive involvement, suggesting that additional biological and systemic factors contribute to heterogeneity in the clinical and neuroimaging correlates of amyloid pathology (Doherty et al., 2015; Bachmann et al., 2023). Identifying factors that modify these associations may therefore help characterize why cerebral Aβ burden is more strongly associated with structural vulnerability in some individuals than in others.

The entorhinal cortex (EC) is particularly relevant to this question because of its vulnerability to AD-related pathological processes and its central role in episodic memory. The EC provides an important interface between the temporal neocortex and hippocampal formation and contributes to the encoding, consolidation, and retrieval of episodic information (Marks et al., 2017; Maass et al., 2018). Lower EC thickness has been associated with impaired episodic memory and AD-related neurodegeneration, while longitudinal studies have also documented progressive EC structural involvement during disease evolution (Knopman et al., 2019; Bachmann et al., 2023; Gagliardi et al., 2023). Nevertheless, the correspondence between cerebral Aβ burden and EC structural integrity is not uniform across individuals (Huijbers et al., 2014; Doherty et al., 2015). This variability provides a rationale for examining systemic factors that may modify the cross-sectional association between Aβ burden and EC thickness and, in turn, its statistical relationship with episodic memory performance.

Circulating lipid status represents one potential systemic context in which such heterogeneity may occur. Although intact peripheral lipoproteins do not readily cross the blood–brain barrier, peripheral lipid measures have been associated with cerebral amyloidosis, AD neuropathology, and vascular-metabolic factors relevant to brain structure (Chen et al., 2014; Reed et al., 2014; Wingo et al., 2022). Previous work has also suggested that vascular risk may interact with Aβ burden in relation to cortical thickness in AD-vulnerable regions (Villeneuve et al., 2014). Low-density lipoprotein cholesterol (LDL-C) is of particular interest because circulating LDL-C has been associated with AD-related biomarkers and structural phenotypes (Akyol et al., 2023; Zhang et al., 2025). Biological studies involving vascular Aβ-clearance pathways, lipoprotein-receptor systems, and cholesterol-dependent membrane processes provide plausible links between lipid metabolism and amyloid-associated brain phenotypes (Ehehalt et al., 2003; Zlokovic et al., 2010; Sagare et al., 2012; Ramanathan et al., 2015). Accordingly, LDL-C was selected as the primary lipid moderator, while other circulating lipid measures were examined in secondary analyses to assess the specificity of the observed association.

Previous studies support the possibility that circulating lipid status modifies amyloid-related brain pathology. Han et al. (2024) reported that blood LDL-C modified the association between cerebral Aβ and tau deposition, indicating that LDL-dependent heterogeneity in AD biomarkers has previously been observed. Separately, previous work within the Gwangju Alzheimer’s Disease and Related Dementias (GARD) resource showed that triglycerides moderated the association between cerebral Aβ burden and hippocampal volume in individuals with MCI and early AD dementia (Seok et al., 2025). The present study addresses a distinct question by focusing on surface-derived EC cortical thickness, domain-specific episodic memory performance, and a broader GARD cohort that includes cognitively normal individuals as well as participants with MCI and AD dementia (Choi et al., 2026). Thus, the present study examined whether circulating LDL-C contributes to heterogeneity in the Aβ–EC–memory relationship.

In this cross-sectional study, we therefore examined the statistical associations among cerebral Aβ burden, EC thickness, LDL-C, and episodic memory performance. First, we tested whether Aβ burden was indirectly associated with episodic memory performance through EC thickness. Second, we tested whether LDL-C modified the Aβ–EC association, hypothesizing that higher LDL-C would be associated with a more negative conditional association between Aβ burden and EC thickness. Third, we evaluated whether the indirect statistical association between Aβ burden and episodic memory through EC thickness varied continuously across LDL-C levels. Secondary analyses evaluated sensitivity to clinical diagnostic adjustment and clinical stage, robustness to model-form and influence assumptions, and whether comparable effect modification was observed for other circulating lipid measures.

## 2 Materials and methods

### 2.1 Study population and design

We analyzed cross-sectional data drawn from the Gwangju Alzheimer’s Disease and Related Dementias (GARD) cohort. This large-scale, prospective, community-based observational study in South Korea was established to comprehensively investigate clinical, cognitive, and neuroimaging biomarkers associated with normative aging and dementia progression (Seo et al., 2021; Choi et al., 2026). Cross-sectional baseline data were used to assess the statistical associations among brain Aβ burden, EC thickness, LDL-C, and episodic memory function at a single point in time. Participants aged ≥ 55 years were eligible for inclusion. Exclusion criteria were established to exclude other conditions and included: (1) structural lesions of the brain as seen on magnetic resonance imaging (MRI) including cerebral infarction, tumor, hydrocephalus, or severe white matter hyperintensities; (2) history of a major neurological or psychiatric disorder; (3) severe sensory or physical impairments precluding standard neuropsychological assessments; (4) current use of psychoactive medications; and (5) abnormal laboratory findings indicative of alternate causes of cognitive impairment (e.g., syphilis, severe vitamin B12 deficiency) (Seok et al., 2025).

Clinical diagnostic labels were obtained from the GARD registry. Diagnostic classification was established by dementia specialists through multistep assessment integrating Clinical Dementia Rating (CDR), comprehensive neuropsychological performance, functional status, medical history, and neuroimaging information (Choi et al., 2026). CN indicated no clinically significant cognitive or functional impairment; MCI indicated objective cognitive impairment without dementia-level functional loss; and AD dementia reflected more global cognitive impairment accompanied by clinically significant functional decline. CDR was therefore considered one component of clinical staging rather than a sole diagnostic rule. Registry labels were harmonized to the three-level CN/MCI/AD classification for the present analyses (Choi et al., 2026).

When multiple eligible records were available for an individual, a deterministic subject-level procedure retained the record with the greatest completeness of prespecified analysis variables; ties were resolved by the earliest visit and then the earliest sequence number. No missing values were imputed. The primary complete-case cohort required available Aβ burden, bilateral EC thickness, low-density lipoprotein cholesterol (LDL-C), episodic memory, age, sex, years of education, body mass index (BMI), and glycated hemoglobin (HbA1c), yielding 1,253 participants. Detailed cohort derivation and modality-specific exclusions are presented in Supplementary Figure 1 and Supplementary Table 1 and are summarized in the Results rather than repeated here. The study was conducted in accordance with the Declaration of Helsinki and was approved by the Institutional Review Board of Chosun University Hospital (Approval No. CHOSUN 2019-10-022-025) and Chonnam National University Hospital (CNUH-2019-279). Written informed consent was obtained from participants or their legally authorized representatives.

### 2.2 Clinical and neuropsychological assessments

Demographic and clinical measures at baseline included age, sex, years of education, body mass index (BMI; kg/m^2^), and systolic and diastolic blood pressure (mmHg). Global cognitive status was evaluated using the Mini-Mental State Examination (MMSE), which was used as a descriptive variable of general cognitive function (Jun et al., 2024; Seok et al., 2025). Episodic memory was assessed using the memory domain score of the Seoul Neuropsychological Screening Battery, Second Edition (SNSB-II), a domain-specific measure sensitive to memory impairment across the aging and AD spectrum (Jun et al., 2024; Ban et al., 2026; Choi et al., 2026). The memory domain of the SNSB-II was used as the principal cognitive measure of interest in both mediation and moderated mediation models. Memory scores were converted to z-scores for regression analyses within the analytic sample.

### 2.3 Blood biomarkers and lipid profiling

Fasting venous blood was used for lipid and metabolic profiling. LDL-C, high-density lipoprotein cholesterol (HDL-C), triglycerides, and total cholesterol were measured using routine enzymatic colorimetric methods with a Modular D2400 analyzer (Roche Diagnostics, Basel, Switzerland), consistent with previous GARD analyses (Seok et al., 2025). HbA1c was included as a metabolic covariate to account for variation in longer-term glycemic status.

Low-density lipoprotein cholesterol was specified as the primary lipid moderator. Secondary moderator analyses examined HDL-C, triglycerides, total cholesterol, and non-HDL cholesterol, with non-HDL cholesterol calculated as total cholesterol minus HDL-C. Log-transformed triglycerides were additionally examined as a transformation sensitivity analysis.

### 2.4 Neuroimaging data acquisition and processing

#### 2.4.1 Structural MRI acquisition and preprocessing

High-resolution T1-weighted structural images were acquired using a 3.0-T Siemens Skyra scanner with a 20-channel head coil and a three-dimensional magnetization-prepared rapid gradient-echo (MPRAGE) sequence (repetition time = 2,300 ms, echo time = 2.143 ms, inversion time = 900 ms, flip angle = 9°, field of view = 256 × 256, acquisition matrix = 320 × 320, and slice thickness = 0.8 mm).

Structural T1-weighted MR images were processed using the standard recon-all pipeline implemented in FreeSurfer version 5.3.0. Briefly, preprocessing included intensity normalization (Sled et al., 1998), segmentation of gray matter (GM), white matter (WM), and cerebrospinal fluid (CSF), and reconstruction of the gray–white matter and pial surfaces (Dale et al., 1999; Fischl et al., 2001). Following cortical surface reconstruction, the surfaces were inflated and registered to a spherical atlas using individual cortical folding patterns to align cortical geometry across participants (Fischl et al., 1999a,b). The cerebral cortex was automatically parcellated according to the Desikan–Killiany–Tourville (DKT) atlas (Klein and Tourville, 2012), which provides anatomically defined cortical regions based on gyral and sulcal landmarks. For each cortical region of interest (ROI), mean cortical thickness and surface area were extracted separately for the left and right hemispheres. Cortical thickness was determined from the distance between the gray–white matter boundary and the pial surface across the reconstructed cortical surface (Fischl and Dale, 2000) and was expressed in millimeters (mm). Regional cortical surface area was expressed in square millimeters. Left and right entorhinal cortical-thickness values were averaged to derive bilateral EC thickness, which served as the structural mediator in the statistical models. Following automated reconstruction, the pial and white-matter surfaces were visually inspected by two trained neuroimaging analysts who were blinded to participants’ clinical status. Scans with gross topological or skull-stripping errors or severe motion-related artifacts were excluded during quality control. Regional mean EC thickness was obtained from the parcellated cortical-thickness data without additional volumetric Gaussian smoothing. Because the structural phenotype of interest was cortical thickness rather than cortical volume or surface area, intracranial volume was not included as a covariate in the primary models.

#### 2.4.2 Amyloid PET acquisition and processing

Cerebral Aβ burden was assessed using florbetaben PET acquired with a Discovery STE PET/CT scanner (GE Healthcare, Milwaukee, WI, USA). Images were obtained approximately 90–100 min after tracer administration.

Cerebral Aβ burden was quantified using BTXBrain-Amyloid software (Brightonix Imaging Inc., Seoul, Republic of Korea), an automated AI-based PET spatial normalization and quantification pipeline, without the requirement of paired MRI. Individual PET activity concentrations were extracted using the Automated Anatomical Labeling (AAL) atlas. Cortical Aβ burden was calculated as a composite standardized uptake value ratio (SUVR) across prespecified target regions—including frontal, lateral temporal, posterior cingulate, and precuneus regions—with the cerebellum gray matter used as the reference region. Global Aβ SUVR was used as the principal independent variable in all subsequent statistical models.

### 2.5 Statistics

All manuscript-facing analyses were performed in R version 4.1.3. Linear regression models were estimated by ordinary least squares; non-parametric bootstrap procedures used the boot package, heteroskedasticity testing used lmtest, and heteroskedasticity-consistent covariance estimates were obtained using sandwich. Regression equations were specified to correspond to Hayes’ PROCESS templates (Hayes, 2012): Model 4 for the unconditional indirect statistical association and Model 7 for first-stage moderated mediation. All mediation terminology refers to cross-sectional indirect statistical associations and does not imply temporal or causal mediation. Analyses were organized hierarchically. Primary models were adjusted for age, sex, years of education, body mass index (BMI), and glycated hemoglobin (HbA1c). Clinical diagnosis was deliberately not included in the primary covariate set because diagnostic classification incorporates cognitive and functional information closely related to the study outcome and may therefore complicate interpretation when conditioned on in models of Aβ, brain structure, and cognition. Diagnosis-adjusted models were instead treated as sensitivity analyses.

Amyloid-beta burden, EC thickness, and LDL-C were expressed on standardized scales for regression modeling, and variables entering interaction terms were mean-centered within the relevant complete-case analysis set before product terms were generated. The SNSB-II memory-domain z-score was used directly as the cognitive outcome. Model 4 and Model 7 were repeated in the same cohort with diagnosis added as a covariate to evaluate model sensitivity.

Model 4 estimated the association between Aβ burden and EC thickness (the a path), the association between EC thickness and memory conditional on Aβ burden (the b path), the direct Aβ–memory association conditional on EC thickness (the c′ path), and the total Aβ–memory association (the c path). The indirect statistical association was calculated as a × b. Statistical uncertainty was estimated using 10,000 non-parametric bootstrap resamples. Bias-corrected (BC) 95% bootstrap confidence intervals were used for primary reporting, with percentile bootstrap intervals additionally examined as sensitivity estimates. Results for which the two interval approaches yielded different conclusions regarding exclusion of zero were treated as interval-method-sensitive evidence.

Model 7 tested whether LDL-C modified the Aβ–EC association by including the mean-centered Aβ × LDL-C product term in the EC-thickness model and evaluated whether the estimated indirect Aβ–memory association through EC thickness varied with LDL-C. The incremental contribution of the interaction was quantified using ΔR^2^ relative to the corresponding model without the interaction and incremental Cohen’s *f*^2^. Conditional Aβ–EC associations and conditional indirect associations were evaluated at the 16th, 50th, and 84th percentiles of the observed LDL-C distribution, with standardized probe values mapped back to mg/dL. Johnson–Neyman analysis was used to characterize regions of the observed LDL-C distribution in which the conditional Aβ–EC association differed statistically from zero; these were interpreted as sample-specific statistical regions of significance rather than clinical thresholds. The index of moderated indirect association was calculated from the product of the Aβ × LDL-C interaction coefficient and the EC–memory coefficient; bootstrap confidence intervals excluding zero were interpreted as statistical support for variation in the indirect association across LDL-C.

Heteroskedasticity was assessed using the Breusch–Pagan test. HC3 covariance estimates were used as the principal inferential standard for the focal Aβ × LDL-C interaction, conditional simple slopes, and Johnson–Neyman analysis, while ordinary least-squares coefficients were retained as point estimates. Model-form robustness was examined by adding quadratic terms for Aβ burden and LDL-C. Influence diagnostics included studentized residuals, leverage, Cook’s distance, and interaction-specific DFBETAS and were treated as screening diagnostics rather than automatic exclusion criteria. Screening thresholds were —studentized residual— > 3, leverage > 2*p*/N, Cook’s D > 4/N, and —DFBETAS— > 2/ N. Stability was evaluated using leave-one-participant-out analysis and, separately, a conservative deletion stress test based on Cook’s D > 4/N or interaction-specific —DFBETAS— > 2/ N. The complete-case full cohort remained the primary analytic sample irrespective of the stress-test result.

Clinical-stage sensitivity analyses repeated Model 7 in CN+MCI, MCI-only, and CN-only subsets and used an omnibus Aβ × LDL-C × diagnosis model to formally evaluate diagnostic heterogeneity. Lipid-specificity analyses fitted parallel moderator models for LDL-C, HDL-C, triglycerides, total cholesterol, and non-HDL cholesterol using both available-case and common lipid-complete samples. Benjamini–Hochberg false-discovery-rate correction was applied to this five-lipid comparison family for conventional and HC3 interaction p-values. Additional analyses adjusted the LDL-C model for triglycerides and evaluated competing Aβ × LDL-C and Aβ × triglyceride interactions; analogous LDL-C/HDL-C models were examined. Where lipid-lowering medication status was available, medication adjustment was compared with an unadjusted reference model fitted to the identical medication-complete subset. Unless otherwise specified, all statistical tests were two-sided with α = 0.05.

## 3 Results

### 3.1 Participant characteristics and analytic cohort

After diagnostic harmonization, eligibility restriction, deterministic subject-level deduplication, and complete-case restriction, the primary analytic cohort comprised 1,253 participants: 798 cognitively normal (CN) participants, 425 participants with mild cognitive impairment (MCI), and 30 participants with AD dementia (Supplementary Figure 1 and Supplementary Table 1). Forty-one participants were excluded from the deduplicated cohort because at least one variable required for the primary analysis was unavailable.

The mean age of the cohort was 72.78 ± 5.98 years, 567 participants (45.3%) were male, and mean education was 12.02 ± 4.62 years. Mean LDL-C was 102.73 ± 35.15 mg/dL and did not differ significantly across diagnostic groups (*p* = 0.477). Cerebral Aβ burden, bilateral entorhinal cortex (EC) thickness, MMSE score, and SNSB-II memory-domain score differed across diagnostic groups (all *p <* 0.001), whereas BMI, triglycerides, fasting glucose, and HbA1c did not show statistically significant group differences (Table 1).

**TABLE 1:** Baseline demographic, clinical, metabolic, neuroimaging, and cognitive characteristics by diagnostic group.

Characteristic	Total	CN	MCI	AD dementia	*P*-value
*N*	1,253	798	425	30	–
Age, years	72.78 ± 5.98	72.37 ± 5.82	73.35 ± 6.08	75.70 ± 7.50	<0.001
Sex, male, *n* (%)	567 (45.3%)	341 (42.7%)	206 (48.5%)	20 (66.7%)	0.009
Education, years	12.02 ± 4.62	11.88 ± 4.59	12.36 ± 4.67	11.13 ± 4.54	0.122
Body mass index, kg/m^2^	24.79 ± 2.91	24.79 ± 2.86	24.82 ± 3.03	24.18 ± 2.44	0.513
Triglycerides, mg/dL^1^	98.00 [74.00, 133.00]	98.00 [75.00, 131.00]	97.00 [71.00, 136.00]	118.50 [94.00, 147.00]	0.124
Total cholesterol, mg/dL	168.21 ± 37.70	169.17 ± 37.22	166.15 ± 38.88	171.87 ± 33.04	0.355
HDL-C, mg/dL	52.53 ± 13.93	53.49 ± 13.77	51.05 ± 14.15	47.79 ± 12.49	0.002
LDL-C, mg/dL	102.73 ± 35.15	103.00 ± 35.09	101.75 ± 35.68	109.47 ± 28.77	0.477
Fasting glucose, mg/dL	73.00 [64.00, 83.00]	72.00 [64.00, 82.00]	74.00 [65.00, 84.00]	70.50 [65.00, 87.75]	0.157
HbA1c, %	5.80 [5.50, 6.10]	5.80 [5.50, 6.10]	5.80 [5.60, 6.20]	5.75 [5.40, 5.90]	0.142
Cerebral Aβ burden, SUVR	1.24 [1.19, 1.36]	1.23 [1.19, 1.33]	1.25 [1.19, 1.41]	1.60 [1.41, 1.71]	<0.001
Bilateral EC thickness, mm	3.46 [3.25, 3.63]	3.50 [3.33, 3.66]	3.38 [3.15, 3.60]	2.91 [2.54, 3.21]	<0.001
MMSE score	28.00 [26.00, 29.00]	28.00 [27.00, 29.00]	27.00 [25.00, 28.00]	21.00 [16.00, 24.00]	<0.001
SNSB-II memory-domain score	−0.03 ± 0.74	0.27 ± 0.58	−0.47 ± 0.62	−1.71 ± 0.50	<0.001

Values are mean ± standard deviation, median (interquartile range), or *n* (%), as appropriate. Group comparisons were performed using one-way analysis of variance, Kruskal–Wallis tests, chi-square tests, or Fisher’s exact tests according to variable distribution and cell size. Aβ, amyloid-beta; AD, Alzheimer’s disease; CN, cognitively normal; EC, entorhinal cortex; HbA1c, glycated hemoglobin; HDL-C, high-density lipoprotein cholesterol; LDL-C, low-density lipoprotein cholesterol; MCI, mild cognitive impairment; MMSE, Mini-Mental State Examination; SNSB-II, Seoul Neuropsychological Screening Battery, Second Edition; SUVR, standardized uptake value ratio.

### 3.2 Primary associations among Aβ burden, EC thickness, and episodic memory

In the primary Model 4, adjusted for age, sex, education, BMI, and HbA1c but not clinical diagnosis, higher Aβ burden was associated with lower bilateral EC thickness (β = −0.0863, SE = 0.0265, 95% CI [−0.1383, −0.0342], *p =* 0.0012). In the episodic-memory outcome model, greater EC thickness was associated with better memory performance after adjustment for Aβ burden and the same covariates (β = 0.3809, SE = 0.0326, 95% CI [0.3170, 0.4448], *p <* 0.001), while greater Aβ burden remained associated with poorer memory performance (β = −0.2173, SE = 0.0306, 95% CI [−0.2774, −0.1571], *p <* 0.001) (Table 2A and Supplementary Table 2).

**TABLE 2:** Primary diagnosis-free and diagnosis-adjusted sensitivity analyses of the Aβ–entorhinal cortex–memory conditional-process models.

Model component	Primary diagnosis-free model, estimate	95% CI/inference	Diagnosis-adjusted sensitivity model, estimate	95% CI/inference
A. Unconditional indirect association (Model 4)				
Aβ→ EC thickness (a path)	−0.0863	SE = 0.0265; 95% CI [−0.1383, −0.0342]; *p* = 0.0012	−0.0162	SE = 0.0259; 95% CI [−0.0671, 0.0347]; *p* = 0.532
EC thickness → memory (b path)	0.3809	SE = 0.0326; 95% CI [0.3170, 0.4448]; *p <* 0.001	0.1800	SE = 0.0281; 95% CI [0.1248, 0.2351]; *p* < 0.001
Direct Aβ→ memory association (c′ path)	−0.2173	SE = 0.0306; 95% CI [−0.2774, −0.1571]; *p* < 0.001	−0.1048	SE = 0.0257; 95% CI [−0.1553, −0.0543]; *p* < 0.001
Indirect statistical association (a × b)	−0.0329	95% BC bootstrap CI [−0.0647, −0.0072]	−0.0029	95% BC bootstrap CI [−0.0163, 0.0080]
B. LDL-C first-stage moderation (Model 7)				
Aβ × LDL-C → EC thickness	−0.0937	HC3 SE = 0.0332; HC3 95% CI [−0.1589, −0.0285]; *p* = 0.0049	−0.0789	HC3 SE = 0.0306; HC3 95% CI [−0.1390, −0.0188]; *p* = 0.0101
ΔR^2^ attributable to Aβ × LDL-C	0.0082	Incremental f^2^ = 0.0094	0.0058	Incremental f^2^ = 0.0074
Index of moderated indirect association	−0.0357	95% BC bootstrap CI [−0.0637, −0.0119]	−0.0142	95% BC bootstrap CI [−0.0281, −0.0038]

*N* = 1,253 for both specifications. Primary models were adjusted for age, sex, years of education, body mass index, and HbA1c; sensitivity models additionally included clinical diagnosis. Focal variables were z-standardized. Ordinary least-squares point estimates are shown; HC3 heteroskedasticity-consistent standard errors and confidence intervals are reported for the Aβ × LDL-C interaction. Indirect associations and the index of moderated indirect association were evaluated using 10,000 non-parametric bootstrap resamples with bias-corrected (BC) 95% confidence intervals. ΔR^2^ is the incremental variance in EC thickness attributable to the Aβ × LDL-C interaction. Because all measures were cross-sectional, indirect associations should not be interpreted as temporal or causal mediation. Aβ, amyloid-beta; BC, bias-corrected; EC, entorhinal cortex; HC3, heteroskedasticity-consistent covariance estimator; LDL-C, low-density lipoprotein cholesterol.

The unconditional indirect statistical association between Aβ burden and episodic memory through EC thickness was β = −0.0329 (BootSE = 0.0145). Both the bias-corrected 95% bootstrap CI [−0.0647, −0.0072] and percentile 95% bootstrap CI [−0.0635, −0.0064] excluded zero.

### 3.3 LDL-C modifies the association between Aβ burden and EC thickness

In Model 7, LDL-C modified the association between Aβ burden and EC thickness. The Aβ × LDL-C interaction was β = −0.0937. Because heteroskedasticity was detected, HC3 covariance estimates were used for primary inference; the interaction remained statistically supported (HC3 SE = 0.0332, 95% CI [−0.1589, −0.0285], *p =* 0.0049). The interaction accounted for an additional ΔR^2^ = 0.00823 in EC thickness, corresponding to an incremental Cohen’s f^2^ = 0.00943 (Table 2B and Supplementary Table 2). Thus, the incremental explanatory contribution of the interaction was small.

Conditional-slope analyses showed that the estimated Aβ–EC association became more negative across increasing LDL-C levels (Figure 1 and Table 3). At the 16th percentile of LDL-C (66 mg/dL; z = −1.0448), the conditional association was positive but not statistically supported (β = 0.0263, HC3 95% CI [−0.0676, 0.1202], *p* = 0.583). At the median (100 mg/dL; z = −0.0819), the estimate was negative but its confidence interval included zero (β = −0.0639, HC3 95% CI [−0.1306, 0.0027], *p* = 0.060). At the 84th percentile (138 mg/dL; z = 0.9942), higher Aβ burden was associated with lower EC thickness (β = −0.1648, HC3 95% CI [−0.2590, −0.0706], *p* < 0.001). Accordingly, the fitted interaction was direction-changing, or crossover in form, but the positive conditional estimate at the lower LDL-C probe was not statistically supported.

**FIGURE 1:**
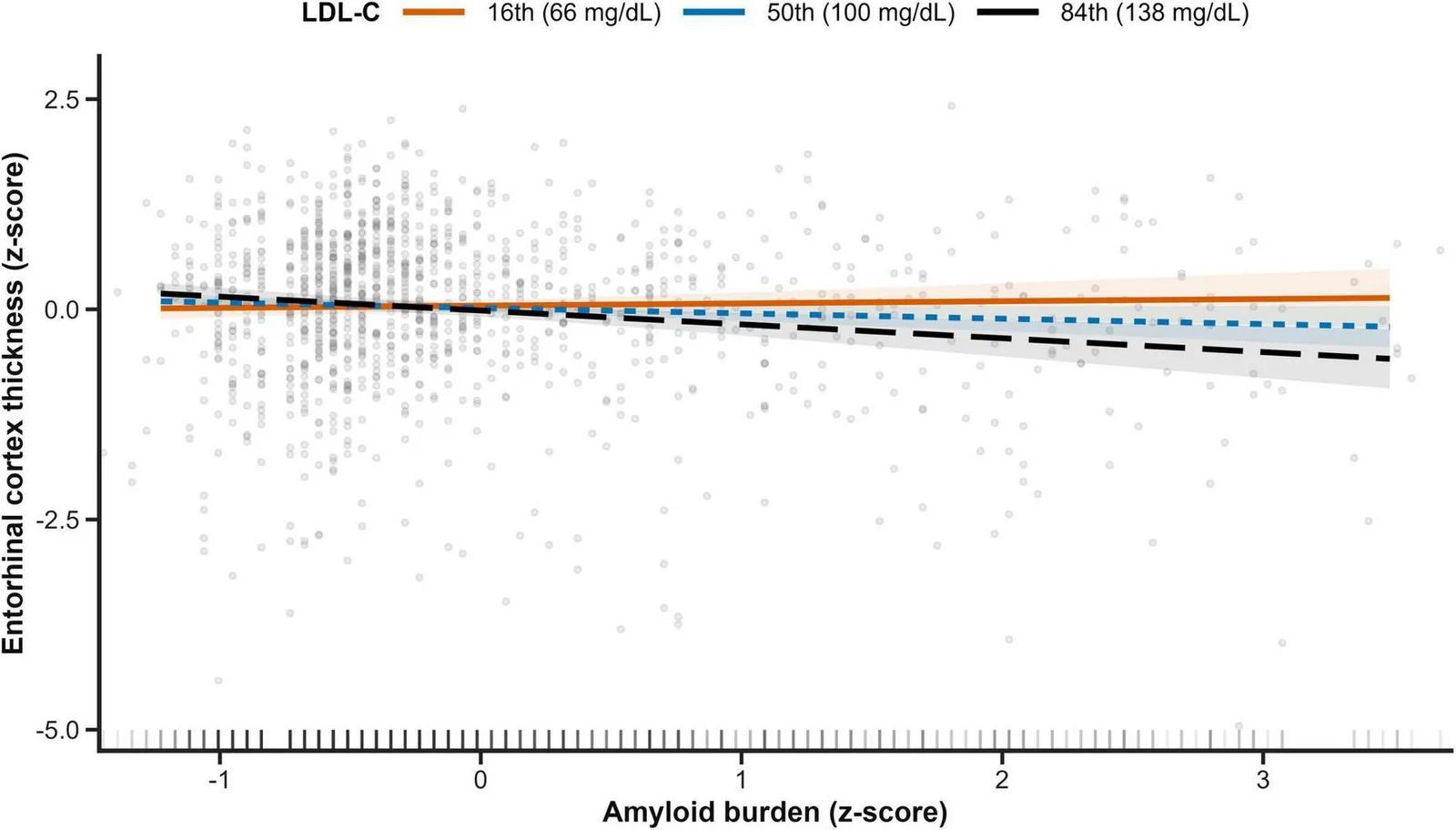
LDL-C-dependent variation in the association between cerebral amyloid burden and entorhinal cortex thickness. Gray points represent observed participant-level values of standardized cerebral amyloid-beta (Aβ) burden and bilateral entorhinal cortex (EC) thickness. Lines represent adjusted ordinary least-squares model predictions at the 16th, 50th, and 84th percentiles of the LDL-C distribution, corresponding to approximately 66, 100, and 138 mg/dL, respectively; shaded bands represent 95% confidence intervals calculated using HC3 heteroskedasticity-consistent covariance estimates. Rug marks along the x-axis show the observed distribution of Aβ burden. The primary model was adjusted for age, sex, years of education, body mass index, and HbA1c, without adjustment for clinical diagnosis. The Aβ × LDL-C interaction was β = −0.0937 (HC3 SE = 0.0332, 95% CI [−0.1589, −0.0285], *p* = 0.0049; ΔR^2^ = 0.0082). The corresponding HC3 conditional Aβ–EC associations were β = 0.0263 at 66 mg/dL (*p* = 0.583), β = −0.0639 at 100 mg/dL (*p* = 0.060), and β = −0.1648 at 138 mg/dL (*p <* 0.001). Although the fitted interaction changed direction across the LDL-C distribution, the positive association estimated at the lower LDL-C probe was not statistically supported. For visual clarity, the displayed Aβ range was restricted to the 1st–99th percentiles; all 1,253 participants were retained in model estimation. Aβ, amyloid-beta; EC, entorhinal cortex; HbA1c, glycated hemoglobin; LDL-C, low-density lipoprotein cholesterol.

**TABLE 3:** Conditional Aβ–entorhinal cortex and indirect Aβ–memory associations across LDL-C levels in the primary model.

LDL-C probe	LDL-C, z-score	LDL-C, mg/dL	Conditional Aβ→ EC association, β (HC3 95% CI)	HC3 *p*-value	Conditional indirect association, β (BootSE)	95% BC bootstrap CI	95% percentile bootstrap CI
Lower, 16th percentile	−1.0448	66	0.0263 [−0.0676, 0.1202]	0.583	0.0100 (0.0178)	[−0.0253, 0.0446]	[−0.0255, 0.0445]
Median, 50th percentile	−0.0819	100	−0.0639 [−0.1306, 0.0027]	0.060	−0.0244 (0.0136)	[−0.0542, −0.0004]	[−0.0529, 0.0005]
Higher, 84th percentile	0.9942	138	−0.1648 [−0.2590, −0.0706]	<0.001	−0.0628 (0.0203)	[−0.1074, −0.0265]	[−0.1051, −0.0251]

Conditional Aβ–EC associations were evaluated using HC3 heteroskedasticity-consistent covariance estimates. Conditional indirect associations and the index of moderated indirect association were estimated using 10,000 bootstrap resamples. Bias-corrected intervals were prespecified for primary reporting, with percentile intervals shown as sensitivity estimates. At the median LDL-C probe, the BC interval narrowly excluded zero whereas the percentile interval included zero; this probe should therefore be interpreted as interval-method sensitive rather than unequivocal evidence of a non-zero conditional indirect association. At the higher LDL-C probe, both bootstrap approaches excluded zero. LDL-C probe values are sample-specific distributional values and should not be interpreted as clinical thresholds. Aβ, amyloid-beta; BC, bias-corrected; EC, entorhinal cortex; HC3, heteroskedasticity-consistent covariance estimator; LDL-C, low-density lipoprotein cholesterol. Index of moderated indirect association: β = −0.0357, BootSE = 0.0131, 95% BC bootstrap CI [−0.0637, −0.0119]; 95% percentile bootstrap CI [−0.0624, −0.0111].

The HC3 Johnson–Neyman analysis identified one boundary within the observed LDL-C range at z = −0.0539, corresponding to approximately 101.0 mg/dL (Figure 2). Above this boundary, the conditional Aβ–EC association was significantly negative; approximately 49.0% of participants had LDL-C values above the boundary. The empirical distributions of Aβ burden and LDL-C are shown in Supplementary Figures 2, 3, respectively, and the conventional-covariance Johnson–Neyman analysis is provided in Supplementary Figure 4. These Johnson–Neyman boundaries represent sample-specific regions of statistical significance rather than clinical LDL-C thresholds.

**FIGURE 2:**
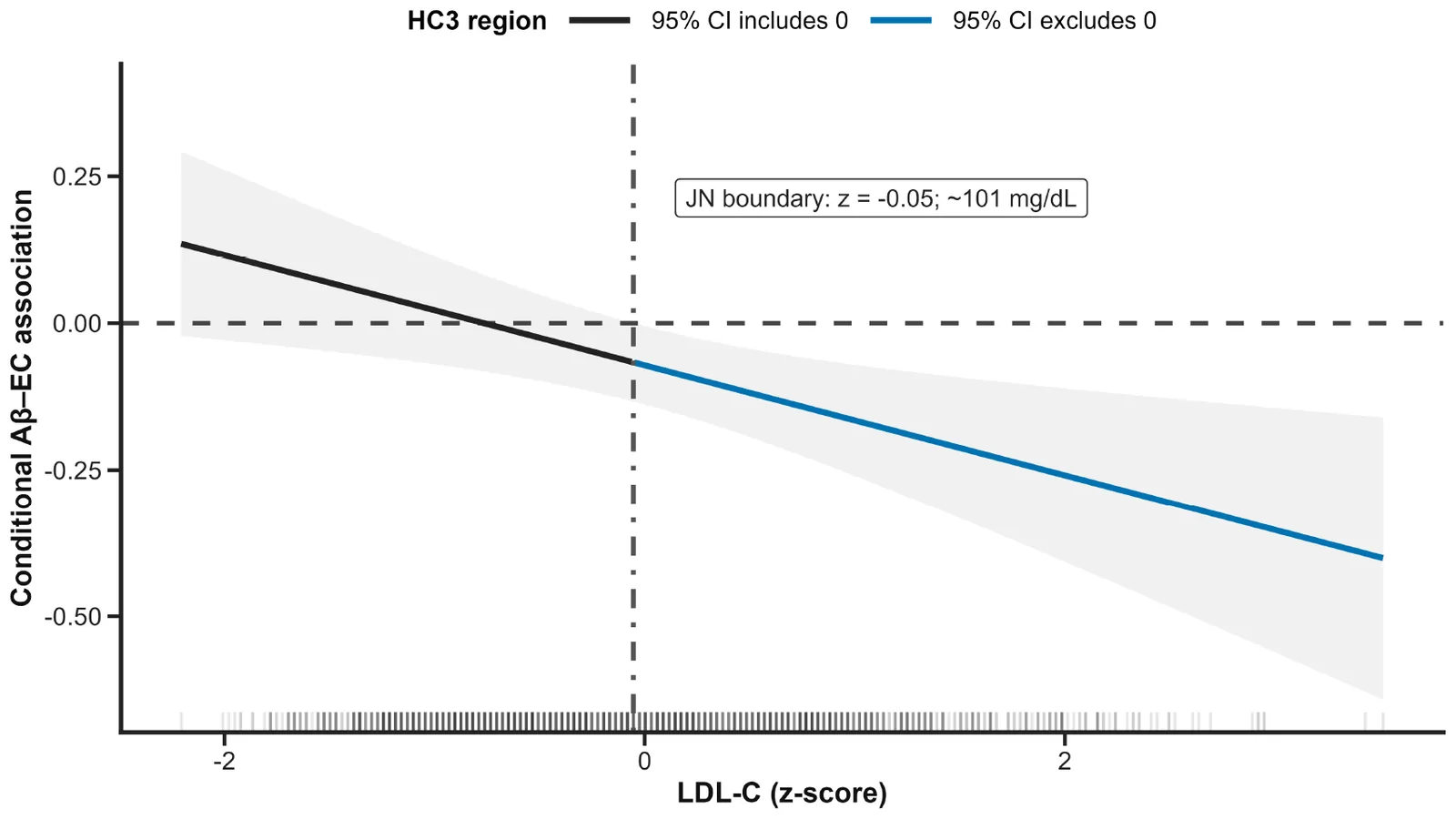
HC3 Johnson–Neyman analysis of the LDL-C-dependent association between cerebral amyloid burden and entorhinal cortex thickness. The solid curve represents the estimated conditional ordinary least-squares association between standardized cerebral amyloid-beta (Aβ) burden and bilateral entorhinal cortex (EC) thickness across the continuous LDL-C distribution. The gray band represents the corresponding 95% confidence interval calculated using HC3 heteroskedasticity-consistent covariance estimates. Curve segments distinguish regions in which the 95% confidence interval includes zero from those in which it excludes zero; the horizontal dashed line denotes a conditional association of zero. Rug marks along the x-axis show the observed LDL-C distribution. The vertical dot-dashed line identifies the Johnson–Neyman boundary within the observed LDL-C range at z = −0.0539, corresponding to approximately 101.0 mg/dL. Above this boundary, the conditional Aβ–EC association was statistically significantly negative under HC3 inference; approximately 49.0% of participants had LDL-C values above this boundary. A second mathematical Johnson–Neyman solution occurred at z = −2.8544 (approximately 2.1 mg/dL), but this value lay outside the observed LDL-C range and is therefore not displayed or interpreted. The model was adjusted for age, sex, years of education, body mass index, and HbA1c, without adjustment for clinical diagnosis. Johnson–Neyman boundaries represent sample-specific statistical regions of significance and should not be interpreted as clinical LDL-C thresholds or treatment targets. Aβ, amyloid-beta; EC, entorhinal cortex; HbA1c, glycated hemoglobin; LDL-C, low-density lipoprotein cholesterol.

### 3.4 LDL-C-dependent indirect statistical association with episodic memory

The index of moderated indirect association was −0.0357 (BootSE = 0.0131), with a bias-corrected 95% bootstrap CI [−0.0637, −0.0119] and percentile 95% bootstrap CI [−0.0624, −0.0111], both excluding zero. This finding indicated that the estimated indirect Aβ–memory association through EC thickness varied as a function of LDL-C (Table 3).

At 66 mg/dL, the conditional indirect association was not statistically supported (β = 0.0100, BootSE = 0.0178; BC 95% CI [−0.0253, 0.0446]; percentile 95% CI [−0.0255, 0.0445]). At 100 mg/dL, the conditional indirect estimate was −0.0244 (BootSE = 0.0136); the bias-corrected interval narrowly excluded zero [−0.0542, −0.0004], whereas the percentile interval included zero [−0.0529, 0.0005], indicating sensitivity to the bootstrap interval method. At 138 mg/dL, the conditional indirect association was negative and supported by both bootstrap approaches (β = −0.0628, BootSE = 0.0203; BC 95% CI [−0.1074, −0.0265]; percentile 95% CI [−0.1051, −0.0251]).

### 3.5 Robustness and sensitivity analyses

#### 3.5.1 Regression diagnostics and model robustness

Variance inflation factors ranged from 1.031 to 1.257. The Breusch–Pagan test indicated heteroskedasticity [χ^2^(8) = 35.25, *p* = 2.40 × 10^–5^], supporting the use of HC3 covariance estimates for the focal interaction (Supplementary Table 5). Adding quadratic terms for Aβ burden and LDL-C did not significantly improve model fit [ΔR^2^ = 0.00258, F(2,1242) = 1.840, *p* = 0.159], while the Aβ × LDL-C interaction remained negative under HC3 inference (β = −0.0849, 95% CI [−0.1487, −0.0212], *p* = 0.0091).

Influence diagnostics identified 73 observations meeting the Cook’s distance screening criterion and 60 meeting the interaction-specific DFBETAS criterion (Supplementary Figure 5 and Supplementary Table 5). Across all 1,253 leave-one-participant-out models, interaction estimates retained the negative direction and ranged from −0.1028 to −0.0810. In contrast, a deliberately conservative stress test excluding 98 participants meeting either screening criterion attenuated the interaction (*N* = 1,155; β = −0.0368, HC3 95% CI [−0.0816, 0.0081], *p =* 0.108), and the confidence interval for the corresponding moderated-indirect index also included zero.

#### 3.5.2 Sensitivity to clinical diagnosis and clinical stage

Adding clinical diagnosis to the same *N* = 1,253 sample attenuated the unconditional associations. The Aβ–EC coefficient changed from −0.0863 (*p =* 0.0012) in the primary diagnosis-free model to −0.0162 (*p =* 0.532) after diagnosis adjustment, and the indirect statistical association changed from −0.0329 (BC 95% CI [−0.0647, −0.0072]) to −0.0029 (BC 95% CI [−0.0163, 0.0080]). In contrast, the focal Aβ × LDL-C interaction remained statistically supported after adjustment for diagnosis (β = −0.0789, HC3 95% CI [−0.1390, −0.0188], *p =* 0.0101; ΔR^2^ = 0.00582), as did the moderated-indirect index (β = −0.0142, BC 95% CI [−0.0281, −0.0038]) (Table 2 and Supplementary Table 3).

In diagnosis-restricted analyses, the Aβ × LDL-C interaction was supported in participants with MCI (*N* = 425; β = −0.1068, HC3 95% CI [−0.2014, −0.0122], *p =* 0.0270) and in the pooled CN+MCI sample (*N* = 1,223; β = −0.0685, HC3 95% CI [−0.1335, −0.0035], *p =* 0.0389), but not in CN participants alone (*N* = 798; β = −0.0090, HC3 95% CI [−0.0883, 0.0704], *p =* 0.824). The corresponding moderated-indirect indices showed the same pattern, with intervals excluding zero in the MCI and pooled CN+MCI analyses but not in the CN-only analysis (Supplementary Table 4). However, the formal Aβ × LDL-C × diagnosis omnibus test was not statistically significant [ΔR^2^ = 0.00177, F(2,1236) = 1.4055, *p =* 0.2456].

#### 3.5.3 Lipid-specificity and competing-lipid analyses

Parallel moderation analyses were conducted in the common lipid-complete sample (*N* = 1,251) (Supplementary Figure 6 and Supplementary Table 6). After HC3 inference and Benjamini–Hochberg correction across the five-lipid comparison family, the Aβ × LDL-C interaction remained statistically supported (β = −0.0934, HC3 95% CI [−0.1587, −0.0282], *p =* 0.0050, q = 0.0251), as did the Aβ × non-HDL-cholesterol interaction (β = −0.0864, HC3 95% CI [−0.1522, −0.0205], *p =* 0.0102, q = 0.0255). Interactions involving HDL-C (*p =* 0.0892, q = 0.1115), triglycerides (*p =* 0.4309, q = 0.4309), and total cholesterol (*p =* 0.0813, q = 0.1115) were not supported under the same HC3/FDR framework. Accordingly, the parallel analyses did not indicate strict specificity of the moderation pattern to LDL-C.

The LDL-C interaction changed little after adjustment for the triglyceride main effect (β = −0.0934, HC3 *p =* 0.0051) and remained statistically supported when the Aβ × LDL-C and Aβ × triglyceride interaction terms were entered simultaneously (β = −0.0923, HC3 95% CI [−0.1584, −0.0263], *p =* 0.0062) (Supplementary Table 7). In the smaller subset with lipid-lowering medication data (*N* = 492), adjustment for medication status produced little change in the interaction point estimate (−0.0502 without medication adjustment versus −0.0526 with adjustment), but both estimates were imprecise and non-significant (HC3 *p =* 0.447 and 0.427, respectively). This subset analysis therefore did not establish robustness of the primary association to lipid-lowering treatment.

## 4 Discussion

In this cross-sectional study of 1,253 older adults across the Alzheimer’s disease (AD) spectrum, we tested whether systemic low-density lipoprotein cholesterol (LDL-C) moderates the statistical association between cerebral amyloid-beta (Aβ) burden, entorhinal cortex (EC) thickness, and episodic memory performance. The primary analysis yielded two complementary findings. First, greater Aβ burden was associated with lower EC thickness. EC thickness was positively associated with episodic memory performance and an unconditional indirect statistical association between Aβ burden and memory through EC thickness was supported. Second, LDL-C further modified the Aβ–EC association: the interaction was negative (β = −0.0937, HC3 *p =* 0.0049) and the estimated association between Aβ burden and EC thickness became increasingly negative toward higher LDL-C levels. The corresponding index of moderated indirect association was also supported (β = −0.0357, 95% bias-corrected bootstrap CI [−0.0637, −0.0119]), indicating that the estimated indirect Aβ–memory association through EC thickness varied across LDL-C levels. Importantly, the interaction accounted for a small increment in explained variance (ΔR^2^ = 0.0082). These findings suggest that circulating lipid status contributes modestly to heterogeneity in the cross-sectional structural correlates of cerebral Aβ burden.

The observed Aβ–EC association is consistent with previous evidence of substantial interindividual variability in the relationship between cerebral amyloid burden, cortical structure, and cognitive performance including within medial temporal and other AD-vulnerable regions (Doherty et al., 2015; Knopman et al., 2019; Bachmann et al., 2023). The EC is particularly relevant in this context because of its central role within medial temporal memory circuitry and its vulnerability to AD-related pathological processes (Marks et al., 2017; Maass et al., 2018). Lower EC thickness and progressive EC thinning have been associated with impaired episodic memory and AD-related neurodegeneration (Knopman et al., 2019; Bachmann et al., 2023; Gagliardi et al., 2023). EC structure may nevertheless vary substantially among individuals with comparable levels of cerebral Aβ (Huijbers et al., 2014; Doherty et al., 2015). In the present analysis, the overall negative Aβ–EC association therefore represents the average association across the cohort, whereas the significant Aβ × LDL-C term indicates additional between-individual variation around that average. LDL-C should consequently be viewed as one potential systemic correlate of this heterogeneity, rather than as a necessary condition for an Aβ–EC association to occur.

The conditional analyses also refine the interpretation of how the Aβ–EC association varies across the LDL-C distribution. At the 16th percentile of LDL-C (approximately 66 mg/dL), the estimated Aβ–EC association was positive but small and not statistically supported (β = 0.0263, HC3 *p* = 0.583). At the median LDL-C level (approximately 100 mg/dL), the estimate was negative but its confidence interval included zero (β = −0.0639, HC3 *p* = 0.060), whereas at the 84th percentile (approximately 138 mg/dL), higher Aβ burden was associated with lower EC thickness (β = −0.1648, HC3 *p* < 0.001). The HC3 Johnson–Neyman analysis similarly identified an in-range statistical boundary at approximately 101 mg/dL, above which the negative conditional association excluded zero. This value should not be interpreted as a biological or treatment threshold; Johnson–Neyman boundaries are sample-specific inferential quantities, and the LDL-C probes used here characterize the observed distribution rather than clinical categories.

The moderated-indirect findings require a similarly differentiated interpretation. The unconditional indirect association and the index of moderated indirect association address related but distinct questions. The former indicates that, under the specified cross-sectional model, Aβ burden was statistically associated with memory performance through EC thickness when averaged across LDL-C levels. The latter indicates that the magnitude of this estimated indirect association changed systematically as a function of LDL-C. Consistent with the first-stage interaction, the conditional indirect association was unsupported at 66 mg/dL. At the median LDL-C probe, the estimate was negative but inferentially sensitive to the bootstrap method: the bias-corrected interval narrowly excluded zero, whereas the percentile interval included zero. At 138 mg/dL, the conditional indirect association was negative and supported by both bootstrap approaches. These findings therefore provide the strongest pointwise evidence toward the higher end of the observed LDL-C distribution, while the significant index provides evidence for continuous variation in the indirect statistical association across LDL-C. Neither result establishes a temporal sequence in which Aβ causes EC degeneration that subsequently causes memory impairment. Because Aβ PET, EC thickness, lipid measures, and cognition were analyzed cross-sectionally, alternative temporal and causal structures remain possible. For this reason, the present findings are described as indirect statistical associations rather than evidence of a demonstrated biological mediation pathway.

Several biological processes provide plausible context for the observed effect modification, although none was directly assessed. Intact peripheral LDL particles do not readily cross the blood–brain barrier, but peripheral lipid metabolism has been associated with cerebral amyloidosis, AD neuropathological burden, and vascular-metabolic risk (Chen et al., 2014; Reed et al., 2014; Wingo et al., 2022). One possible context involves vascular and Aβ-clearance processes. Low-density lipoprotein receptor-related protein 1 (LRP1) and related transport mechanisms participate in Aβ clearance at the neurovascular interface, and disturbances in vascular-mediated clearance have been implicated in AD biology (Zlokovic et al., 2010; Sagare et al., 2012; Ramanathan et al., 2015). A separate possibility is that systemic lipid alterations covary with changes in central lipid homeostasis or cholesterol-enriched membrane microdomains involved in amyloid precursor protein processing, Aβ generation, and aggregation (Ehehalt et al., 2003; Rushworth and Hooper, 2010; Rudajev and Novotny, 2022). These mechanisms provide biological plausibility for the observed association but were not directly assessed in the present study. In addition, tau pathology was not incorporated into the present analysis, which is particularly relevant because entorhinal structural change and episodic memory impairment are closely associated with tau accumulation (Marks et al., 2017; Maass et al., 2018).

The lipid-specificity analyses further qualify the biological interpretation. Previous neuroimaging studies have linked vascular or metabolic risk to the relationship between cerebral Aβ and cortical structure (Villeneuve et al., 2014). More recent studies have related circulating lipid measures to AD biomarkers and brain structural phenotypes (Kang et al., 2023; Han et al., 2024; Zhang et al., 2025). Consistent with prior reports linking circulating lipids to AD biomarkers, the present study found that both LDL-C and non-HDL cholesterol modified the Aβ–EC association (Han et al., 2024). The additional lipid analyses also argue against strict LDL-C specificity. In a common lipid-complete sample, both LDL-C and non-HDL cholesterol interactions remained supported after HC3 inference and Benjamini–Hochberg false-discovery-rate correction, whereas the corresponding HDL-C, triglyceride, and total-cholesterol interactions were not supported under the same framework. The LDL-C interaction additionally changed little after adjustment for triglycerides and remained supported when Aβ × LDL-C and Aβ × triglyceride interactions were included simultaneously. Collectively, these findings suggest that the observed moderation may be more appropriately considered within a broader atherogenic-cholesterol context, while LDL-C remains the prespecified primary moderator examined in the present study.

Sensitivity to diagnostic adjustment provides additional information about the statistical structure of the findings. Clinical diagnosis was excluded from the primary models because diagnostic classification incorporates cognitive and functional information closely related to the outcome and could therefore complicate interpretation when conditioned upon. When diagnosis was added to the identical *N* = 1,253 sample, the unconditional Aβ–EC association and indirect statistical association were markedly attenuated and no longer statistically supported. In contrast, the Aβ × LDL-C interaction remained supported after diagnosis adjustment (β = −0.0789, HC3 *p* = 0.0101), as did the moderated-indirect index. These comparisons indicate that estimates of the unconditional Aβ–EC–memory associations are sensitive to conditioning on clinical diagnosis, whereas the focal interaction is less dependent on this adjustment. However, these analyses do not establish that diagnosis adjustment itself creates collider bias or statistical suppression; rather, they demonstrate that the estimated associations are sensitive to whether clinical diagnosis is included in the model. The clinical-stage analyses should also be interpreted cautiously. The interaction and moderated-indirect index were supported in MCI and in the pooled CN+MCI analysis, but not in CN alone. Nevertheless, differences in subgroup statistical significance do not establish differences between groups, and the formal Aβ × LDL-C × diagnosis test did not detect statistically significant heterogeneity (ΔR^2^ = 0.00177, *p* = 0.2456). Given that only 30 participants had AD dementia, the non-significant omnibus interaction should not be regarded as evidence that the association is equivalent across clinical stages.

The broader robustness analyses likewise support a qualified rather than absolute statement of stability. Heteroskedasticity was present, but the focal interaction remained supported using HC3 heteroskedasticity-consistent inference. Addition of quadratic Aβ and LDL-C terms did not significantly improve model fit, and the Aβ × LDL-C interaction remained negative and statistically supported in the expanded model, arguing against simple curvature as a sufficient explanation for the primary interaction. Moreover, all 1,253 leave-one-participant-out estimates retained a negative interaction coefficient, indicating that no single participant determined the direction of the finding. However, the deliberately conservative stress test that simultaneously excluded 98 observations meeting either Cook’s distance or interaction-specific DFBETAS screening criteria attenuated the interaction, with the HC3 confidence interval including zero. The corresponding moderated-indirect index was also attenuated. These results argue against dependence on one isolated influential observation but indicate sensitivity to the aggregate influence of a subset of observations. Accordingly, the finding should not be described as uniformly robust across all analytic perturbations. Rather, it was supported across several complementary specifications while showing attenuation under a deliberately broad multi-observation deletion stress test.

The present study should also be interpreted in relation to our previous GARD analysis of triglycerides, Aβ burden, hippocampal volume, and global cognition (Seok et al., 2025). All 188 participants in that earlier analysis are included in the present cohort and represent approximately 15.0% of the current *N* = 1,253 analytic sample. Thus, the two studies draw partly on the same underlying GARD resource and the present analysis should not be regarded as an independent cohort replication. However, they address related but distinct scientific questions. The previous study focused on triglyceride-dependent variation in an Aβ–hippocampal volume–global cognition model among cognitively impaired participants, whereas the present study evaluates LDL-C-dependent variation in the association of cerebral Aβ with EC cortical thickness and domain-specific episodic memory across a substantially broader cohort that includes a large CN group. Moreover, in the current common-sample lipid analyses, triglycerides did not show evidence of the same moderation pattern observed for LDL-C. The present work therefore extends the broader question of metabolic heterogeneity in amyloid-related structural associations to a different lipid exposure, an AD-vulnerable entorhinal phenotype, and a domain-specific memory outcome, while remaining a distinct analysis conducted within the shared GARD resource.

Several limitations should be considered. First, the cross-sectional design precludes conclusions regarding temporal ordering or causality. Longitudinal studies with repeated amyloid, MRI, lipid, and cognitive assessments are required to determine whether lipid status predicts subsequent differences in EC structural change or episodic memory trajectories in the context of Aβ burden. Second, the Aβ × LDL-C interaction explained only a small increment in EC-thickness variance (ΔR^2^ = 0.0082), limiting claims regarding clinical effect magnitude. Third, the conservative influence-deletion analysis attenuated the focal interaction, indicating that replication is needed to establish its stability. Fourth, lipid-lowering medication information was incomplete. In the medication-complete subset (*N* = 492), adjustment for lipid-lowering treatment produced little change in the interaction point estimate, but both adjusted and unadjusted estimates were imprecise and non-significant; this analysis therefore cannot establish robustness to treatment effects. Fifth, APOE genotype was not available for inclusion in the present analysis, preventing evaluation of potential APOE-related confounding or effect modification. Sixth, tau biomarkers were not incorporated; consequently, the analysis cannot distinguish a direct amyloid-related structural association from one operating partly through tau-associated neurodegeneration. Seventh, the small AD dementia subgroup substantially limits stage-specific inference. Finally, the complete-case analysis was conducted in a Korean aging cohort, and generalizability to populations with different genetic backgrounds, vascular risk profiles, treatment exposures, or demographic characteristics remains to be established. The Johnson–Neyman boundary should likewise be regarded as a sample-specific statistical region rather than a clinical LDL-C treatment target.

The study also has several strengths. It integrates amyloid PET, surface-derived EC cortical thickness, domain-specific episodic memory assessment, and systemic lipid measurements in a relatively large multimodal cohort comprising predominantly CN and MCI participants together with a smaller AD dementia subgroup. Taken together, the findings indicate that greater cerebral Aβ burden is associated with lower EC thickness and poorer episodic memory performance, while circulating LDL-C contributes modest additional heterogeneity to the Aβ–EC association, with more negative conditional associations observed at higher LDL-C levels. The corresponding indirect statistical association with memory also varied across LDL-C levels. The parallel non-HDL finding suggests that this pattern may reflect a broader atherogenic lipid context rather than an effect unique to LDL-C. Longitudinal studies incorporating tau, genetic susceptibility, treatment exposure, and repeated structural and cognitive measurements are needed to determine the temporal and clinical relevance of these associations.

## Data Availability

The data supporting the findings of this study are not publicly available due to institutional and ethical restrictions. Access to the data may be granted upon reasonable request to the corresponding author, subject to approval by the Gwangju Alzheimer’s and Related Dementias (GARD) cohort and completion of an appropriate data-use agreement.
