# Supplementary material for: Low-density lipoprotein cholesterol modulates amyloid-related entorhinal vulnerability and episodic memory performance in older adults
Source: Front Aging Neurosci. 2026 Sep 24;18:1933036. doi: 10.3389/fnagi.2026.1933036 (PMC13645760; doi:10.3389/fnagi.2026.1933036)
Supplement: Supplementary file 1 [file Table_1.docx]

**SUPPLEMENTARY TABLES**

**Supplementary Table S1. Cohort derivation and participant flow for the primary and clinical-stage sensitivity analyses**

| **Processing step** | **Records/rows** | **Unique participants** | **Description** |
| --- | --- | --- | --- |
| Source dataset | 1,328 | 1,327 | Source records loaded from the GARD analytic dataset. |
| After diagnostic harmonization and eligibility restriction | 1,295 | 1,294 | Diagnostic labels were harmonized to CN, MCI, or AD dementia and restricted the sample to participants aged ≥55 years. |
| After deterministic subject-level deduplication | 1,294 | 1,294 | One record per participant was retained using the prespecified rule prioritizing completeness of key variables and then the earliest eligible visit/sequence. |
| Primary diagnosis-free complete-case cohort | 1,253 | 1,253 | Required complete Aβ burden, bilateral EC thickness, LDL-C, episodic memory, age, sex, education, BMI, and HbA1c data. Final diagnostic composition: CN = 798, MCI = 425, AD dementia = 30. |
| CN+MCI sensitivity cohort | 1,223 | 1,223 | AD dementia participants excluded; diagnosis was not included as a model covariate. |
| CN-only sensitivity cohort | 798 | 798 | Diagnosis-stratified sensitivity analysis. |
| MCI-only sensitivity cohort | 425 | 425 | Diagnosis-stratified sensitivity analysis. |

| **Processing step / exclusion reason** | **Records/rows** | **Unique participants** |
| --- | --- | --- |
| **After deterministic subject-level deduplication** | 1,294 | 1,294 |
| **Excluded before primary complete-case analysis** | 41 | 41 |
| Missing imaging data | — | 9 |
| Missing laboratory data | — | 20 |
| Missing cognitive data | — | 12 |
| Missing demographic/anthropometric covariate data | — | 28 |
| Missing data in >1 modality | — | 10 |
| **Primary diagnosis-free complete-case cohort** | 1,253 | 1,253 |

**Note:** Forty-one participants were excluded between the deduplicated cohort (N = 1,294) and the primary complete-case cohort (N = 1,253) because at least one required primary-analysis variable was unavailable. CN = cognitively normal; MCI = mild cognitive impairment; AD = Alzheimer's disease; Aβ = amyloid-beta; BMI = body mass index; EC = entorhinal cortex; HbA1c = glycated hemoglobin; LDL-C = low-density lipoprotein cholesterol.

## **Supplementary Table S2. Full coefficient estimates for the primary diagnosis-free indirect-association and moderated indirect-association models**

| **Panel A. Model 4: EC-thickness model** | | | | |
| --- | --- | --- | --- | --- |
| **Predictor** | **β** | **SE** | **95% CI** | **p-value** |
| Aβ burden | −0.0863 | 0.0265 | [−0.1383, −0.0342] | 0.0012 |
| Age | −0.0546 | 0.0047 | [−0.0637, −0.0455] | <0.001 |
| Sex, male | 0.0578 | 0.0592 | [−0.0584, 0.1740] | 0.330 |
| Education, years | −0.0092 | 0.0063 | [−0.0217, 0.0032] | 0.147 |
| BMI | −0.0052 | 0.0092 | [−0.0232, 0.0129] | 0.574 |
| HbA1c | −0.0812 | 0.0378 | [−0.1552, −0.0071] | 0.0318 |
| **Model R² = 0.1187.** | | | | |

| **Panel B. Model 4: episodic-memory outcome model** | | | | |
| --- | --- | --- | --- | --- |
| **Predictor** | **β** | **SE** | **95% CI** | **p-value** |
| Aβ burden | −0.2173 | 0.0306 | [−0.2774, −0.1571] | <0.001 |
| EC thickness | 0.3809 | 0.0326 | [0.3170, 0.4448] | <0.001 |
| Age | 0.0186 | 0.0056 | [0.0076, 0.0297] | 0.0010 |
| Sex, male | −0.3242 | 0.0682 | [−0.4579, −0.1905] | <0.001 |
| Education, years | −0.0140 | 0.0073 | [−0.0283, 0.0004] | 0.0560 |
| BMI | 0.0154 | 0.0106 | [−0.0053, 0.0361] | 0.145 |
| HbA1c | −0.0464 | 0.0435 | [−0.1318, 0.0389] | 0.286 |
| **Model R² = 0.1659** | | | | |

**Unconditional indirect statistical association:** β = −0.0329, BootSE = 0.0145, 95% BC bootstrap CI [−0.0647, −0.0072]; percentile bootstrap CI [−0.0635, −0.0064].

### **Panel C. Model 7: EC-thickness model with LDL-C moderation**

| **Predictor** | **β** | **HC3 SE** | **HC3 95% CI** | **HC3 p-value** |
| --- | --- | --- | --- | --- |
| Aβ burden | −0.0712 | 0.0339 | [−0.1378, −0.0046] | 0.0361 |
| LDL-C | −0.0286 | 0.0271 | [−0.0818, 0.0246] | 0.291 |
| Aβ × LDL-C | **−0.0937** | **0.0332** | **[−0.1589, −0.0285]** | **0.0049** |
| Age | −0.0559 | 0.0052 | [−0.0661, −0.0458] | <0.001 |
| Sex, male | 0.0662 | 0.0606 | [−0.0528, 0.1852] | 0.275 |
| Education, years | −0.0100 | 0.0066 | [−0.0228, 0.0029] | 0.129 |
| BMI | −0.0040 | 0.0096 | [−0.0229, 0.0148] | 0.674 |
| HbA1c | −0.0865 | 0.0401 | [−0.1651, −0.0079] | 0.0311 |
| **Model R² = 0.1274; Aβ × LDL-C ΔR² = 0.00823; incremental Cohen's f² = 0.00943.** | | | | |

**Index of moderated indirect association:** β = −0.0357, BootSE = 0.0131, 95% BC bootstrap CI [−0.0637, −0.0119]; percentile bootstrap CI [−0.0624, −0.0111].

**Note:** N = 1,253. All models were adjusted for age, sex, years of education, BMI, and HbA1c; clinical diagnosis was not included in the primary models. Aβ burden, EC thickness, LDL-C, and episodic memory were expressed as z-scores. Model 4 coefficients are conventional OLS estimates. In Panel C, OLS point estimates are shown with HC3 heteroskedasticity-consistent standard errors and confidence intervals. Indirect associations were estimated using 10,000 bootstrap resamples; bias-corrected intervals were the primary bootstrap intervals. Because the analyses are cross-sectional, the indirect associations should not be interpreted as evidence of temporal or causal mediation.

## **Supplementary Table S3. Sensitivity of the indirect and moderated indirect associations to adjustment for clinical diagnosis**

### **Panel A. Diagnosis-adjusted Model 4**

| **Effect** | **β** | **SE/BootSE** | **95% CI** | **Inference** |
| --- | --- | --- | --- | --- |
| Aβ → EC thickness | −0.0162 | 0.0259 | [−0.0671, 0.0347] | *p =* 0.532 |
| EC thickness → memory | 0.1800 | 0.0281 | [0.1248, 0.2351] | *p <* 0.001 |
| Direct Aβ → memory | −0.1048 | 0.0257 | [−0.1553, −0.0543] | *p <* 0.001 |
| Indirect statistical association | −0.0029 | — | BC bootstrap CI [−0.0163, 0.0080] | CI includes 0 |

### **Panel B. Diagnosis-adjusted Model 7**

| **Quantity** | **Estimate** | **Inference** |
| --- | --- | --- |
| Aβ × LDL-C interaction | −0.0789 | HC3 SE = 0.0306; HC3 95% CI [−0.1390, −0.0188]; *p =* 0.0101 |
| ΔR² attributable to interaction | 0.00582 | — |
| Incremental Cohen's f² | 0.00739 | — |
| Index of moderated indirect association | −0.0142 | 95% BC bootstrap CI [−0.0281, −0.0038] |

### **Panel C. Conditional indirect associations in the diagnosis-adjusted Model 7**

| **LDL-C probe** | **mg/dL** | **Conditional indirect β** | **BootSE** | **95% BC bootstrap CI** | **95% percentile CI** |
| --- | --- | --- | --- | --- | --- |
| 16th percentile | 66 | 0.0139 | 0.0084 | [−0.0012, 0.0324] | [−0.0024, 0.0312] |
| 50th percentile | 100 | 0.0003 | 0.0059 | [−0.0125, 0.0110] | [−0.0122, 0.0111] |
| 84th percentile | 138 | −0.0150 | 0.0087 | [−0.0352, −0.0007] | [−0.0338, 0.0002] |

**Note:** N = 1,253. Models included the primary covariates plus dummy-coded clinical diagnosis, with CN as the reference category. Comparison with the diagnosis-free primary model therefore reflects covariate adjustment rather than a change in sample composition. The high-LDL conditional indirect estimate was supported by the bias-corrected interval but was interval-method sensitive because its percentile bootstrap interval included zero. Diagnosis adjustment markedly attenuated the unconditional Aβ–EC and EC–memory associations but did not eliminate the focal Aβ × LDL-C interaction or the moderated-indirect index.

## **Supplementary Table S4. Clinical-stage sensitivity and formal diagnostic-heterogeneity analyses**

### **Panel A. Diagnosis-restricted Model 7 analyses**

| **Analysis** | **N** | **Aβ × LDL-C β** | **HC3 SE** | **HC3 95% CI** | **HC3 p** | **ΔR²** | **Index of moderated indirect association, β (95% BC CI)** |
| --- | --- | --- | --- | --- | --- | --- | --- |
| CN only | 798 | −0.0090 | 0.0404 | [−0.0883, 0.0704] | 0.824 | 0.00008 | −0.0009 [−0.0114, 0.0063] |
| MCI only | 425 | −0.1068 | 0.0481 | [−0.2014, −0.0122] | 0.0270 | 0.01154 | −0.0259 [−0.0535, −0.0042] |
| CN+MCI pooled | 1,223 | −0.0685 | 0.0331 | [−0.1335, −0.0035] | 0.0389 | 0.00465 | −0.0202 [−0.0416, −0.0025] |

### **Panel B. Formal test of diagnostic-stage heterogeneity**

| **Test** | **ΔR²** | **F** | **df** | **p-value** |
| --- | --- | --- | --- | --- |
| Aβ × LDL-C × diagnosis omnibus interaction | 0.00177 | 1.4055 | 2, 1236 | 0.2456 |

**Note:** All subgroup models retained the primary covariates. The MCI-only interaction and moderated-indirect index were statistically supported, whereas the corresponding CN-only estimates were not. These differing subgroup significance patterns do **not** themselves establish diagnostic-stage heterogeneity. The formal Aβ × LDL-C × diagnosis omnibus test did not detect statistically significant heterogeneity. Given the small AD dementia subgroup (n = 30), the nonsignificant omnibus interaction should not be interpreted as evidence of equivalence across stages. The formal omnibus result is ΔR² = 0.00177, F(2,1236) = 1.4055, *p =* 0.2456.

## **Supplementary Table S5. Regression diagnostics and robustness analyses for the primary Aβ × LDL-C interaction**

### **Panel A. Model diagnostics**

| **Diagnostic** | **Result** |
| --- | --- |
| Breusch–Pagan test | χ²(8) = 35.25, *p =* 2.40 × 10⁻⁵ |
| VIF range | 1.031–1.257 |
| Residual skewness | −0.871 |
| Residual excess kurtosis | 2.124 |

The significant Breusch–Pagan result provides the statistical justification for making HC3 the principal interaction inference. VIF values were uniformly low.

### **Panel B. Robustness of the focal Aβ × LDL-C interaction**

| **Analysis** | **N** | **β** | **SE** | **95% CI** | **p-value** |
| --- | --- | --- | --- | --- | --- |
| Primary conventional OLS inference | 1,253 | −0.0937 | 0.0274 | [−0.1474, −0.0400] | <0.001 |
| Primary HC3 inference | 1,253 | −0.0937 | 0.0332 | [−0.1589, −0.0285] | 0.0049 |
| Quadratic Aβ and LDL-C terms added, conventional | 1,253 | −0.0849 | 0.0277 | [−0.1393, −0.0305] | 0.0022 |
| Quadratic model, HC3 inference | 1,253 | −0.0849 | 0.0325 | [−0.1487, −0.0212] | 0.0091 |
| Conservative influence-deletion stress test, HC3 | 1,155 | −0.0368 | 0.0229 | [−0.0816, 0.0081] | 0.108 |

Adding quadratic terms increased R² from 0.12740 to 0.12998, but the quadratic terms did not significantly improve model fit: ΔR² = 0.00258, F(2,1242) = 1.840, *p =* 0.159.

### **Panel C. Influence and leave-one-out stability**

| **Diagnostic** | **Result** |
| --- | --- |
| Studentized-residual flags | 12 |
| Leverage flags | 80 |
| Cook's D flags | 73 |
| Interaction-specific DFBETAS flags | 60 |
| Any diagnostic flag | 144 |
| Cook's D OR DFBETAS stress-test exclusions | 98 (7.82%) |
| Successful leave-one-out models | 1,253/1,253 |
| Minimum leave-one-out interaction estimate | −0.1028 |
| Maximum leave-one-out interaction estimate | −0.0810 |
| Maximum relative single-case change | 13.62% |
| Leave-one-out estimates retaining primary negative sign | 100% |

**Note:** Influence thresholds were used as screening diagnostics, not automatic data-cleaning criteria. The full N = 1,253 cohort remained the primary analytic sample. The conservative deletion analysis intentionally combined Cook's D > 4/N and |DFBETAS| > 2/√N and should therefore be interpreted as a stress test rather than an alternative preferred cohort. After this broad deletion, the interaction and moderated-indirect index were attenuated and their intervals included zero.

## **Supplementary Table S6. Parallel lipid-moderator analyses assessing specificity of the Aβ–entorhinal cortex interaction**

### **Panel A. Common lipid-complete sample**

| **Moderator** | **N** | **Aβ × lipid β** | **HC3 SE** | **HC3 95% CI** | **HC3 p** | **BH-FDR q** | **ΔR²** | **Moderated-indirect index, β (95% BC CI)** |
| --- | --- | --- | --- | --- | --- | --- | --- | --- |
| LDL-C | 1,251 | −0.0934 | 0.0332 | [−0.1587, −0.0282] | 0.0050 | **0.0251** | 0.00820 | −0.0356 [−0.0622, −0.0103] |
| HDL-C | 1,251 | 0.0540 | 0.0318 | [−0.0083, 0.1163] | 0.0892 | 0.1115 | 0.00334 | 0.0206 [−0.0023, 0.0465] |
| Triglycerides | 1,251 | −0.0216 | 0.0274 | [−0.0753, 0.0321] | 0.4309 | 0.4309 | 0.00044 | −0.0082 [−0.0293, 0.0132] |
| Total cholesterol | 1,251 | −0.0587 | 0.0337 | [−0.1247, 0.0073] | 0.0813 | 0.1115 | 0.00318 | −0.0224 [−0.0495, 0.0017] |
| Non-HDL cholesterol | 1,251 | −0.0864 | 0.0336 | [−0.1522, −0.0205] | 0.0102 | **0.0255** | 0.00695 | −0.0329 [−0.0612, −0.0085] |

### **Secondary triglyceride transformation sensitivity**

| **Moderator** | **N** | **Aβ × lipid β** | **HC3 SE** | **HC3 95% CI** | **HC3 p** | **ΔR²** | **Moderated-indirect index, β (95% BC CI)** |
| --- | --- | --- | --- | --- | --- | --- | --- |
| Log-transformed triglycerides | 1,251 | −0.0255 | 0.0278 | [−0.0800, 0.0291] | 0.360 | 0.00066 | −0.0097 [−0.0314, 0.0110] |

**Note:** All moderator variables were z-standardized before model fitting. Models used the identical N = 1,251 lipid-complete sample to prevent sample-composition differences from being mistaken for moderator-specific effects. HC3 heteroskedasticity-consistent inference is reported for interaction terms. Benjamini–Hochberg false-discovery-rate correction was applied to the prespecified five-lipid family comprising LDL-C, HDL-C, triglycerides, total cholesterol, and non-HDL cholesterol. Log-transformed triglycerides were treated as a secondary transformation sensitivity and were not included in the primary multiplicity family. The parallel non-HDL finding indicates that the data do not support a claim of strict LDL-C specificity and may instead be consistent with a broader atherogenic-cholesterol context.

## **Supplementary Table S7. Competing-lipid and lipid-lowering medication sensitivity analyses**

### **Panel A. Stability of the LDL-C interaction after accounting for other lipid fractions**

| **Model** | **N** | **Aβ × LDL-C β** | **HC3 SE** | **HC3 95% CI** | **HC3 p** | **ΔR²** | **Moderated-indirect index, β (95% BC CI)** |
| --- | --- | --- | --- | --- | --- | --- | --- |
| Primary LDL-C model | 1,253 | −0.0937 | 0.0332 | [−0.1589, −0.0285] | 0.0049 | 0.00823 | −0.0357 [−0.0637, −0.0119] |
| LDL-C moderator + triglyceride main effect | 1,251 | −0.0934 | 0.0333 | [−0.1587, −0.0281] | 0.0051 | 0.00819 | −0.0356 [−0.0633, −0.0108] |
| Competing Aβ × LDL-C and Aβ × triglyceride interactions | 1,251 | −0.0923 | 0.0337 | [−0.1584, −0.0263] | 0.0062 | 0.00796 | −0.0352 [−0.0635, −0.0107] |
| LDL-C moderator + HDL-C main effect | 1,253 | −0.0938 | 0.0333 | [−0.1590, −0.0285] | 0.0049 | 0.00823 | −0.0357 [−0.0641, −0.0115] |
| Competing Aβ × LDL-C and Aβ × HDL-C interactions | 1,253 | −0.0940 | 0.0340 | [−0.1607, −0.0274] | 0.0057 | 0.00829 | −0.0358 [−0.0647, −0.0114] |

### **Panel B. Exploratory three-lipid competing-interaction model**

| **Interaction term** | **β** | **SE** | **95% CI** | **Conventional p** |
| --- | --- | --- | --- | --- |
| Aβ × LDL-C | −0.0943 | 0.0274 | [−0.1481, −0.0404] | <0.001 |
| Aβ × HDL-C | 0.0573 | 0.0269 | [0.0044, 0.1102] | 0.0336 |
| Aβ × triglycerides | 0.0086 | 0.0297 | [−0.0496, 0.0668] | 0.772 |

**Note:** Panel B is exploratory and reports the conventional OLS estimates exported by the v9.2 pipeline. It should not receive the same inferential prominence as the prespecified HC3/FDR lipid-comparison family.

### **Panel C. Lipid-lowering medication same-sample sensitivity**

| **Model** | **N** | **Aβ × LDL-C β** | **HC3 SE** | **HC3 95% CI** | **HC3 p** | **ΔR²** | **Moderated-indirect index, β (95% BC CI)** |
| --- | --- | --- | --- | --- | --- | --- | --- |
| Same-sample reference without medication covariate | 492 | −0.0502 | 0.0660 | [−0.1799, 0.0795] | 0.447 | 0.00204 | −0.0161 [−0.0663, 0.0179] |
| Adjusted for lipid-lowering medication | 492 | −0.0526 | 0.0662 | [−0.1827, 0.0774] | 0.427 | 0.00223 | −0.0168 [−0.0660, 0.0181] |

**Note:** Medication data were incomplete, so both medication-adjusted and reference models were fitted in the **same N = 492 subset** to distinguish covariate adjustment from sample-selection effects. Adjustment produced little change in the point estimate, but both estimates were imprecise and nonsignificant; consequently, this analysis should not be described as demonstrating robustness to lipid-lowering therapy.

# **SUPPLEMENTARY FIGURES**

**Supplementary Figure S1. Participant flow for the primary and clinical-stage sensitivity analyses.**

**Note:** The diagram summarizes derivation of the analytic cohort from the source GARD dataset. The source contained 1,328 records representing 1,327 unique participants. Following diagnostic harmonization and eligibility restriction, 1,294 unique participants remained; deterministic subject-level deduplication retained one record for each participant. Complete-case restriction for cerebral amyloid-beta (Aβ) burden, bilateral entorhinal cortex thickness, LDL-C, episodic memory, age, sex, education, body mass index, and HbA1c yielded the primary diagnosis-free cohort of 1,253 participants (CN, n = 798; MCI, n = 425; AD dementia, n = 30). Clinical-stage sensitivity samples were derived as parallel subsets of this primary cohort: CN+MCI, n = 1,223; CN only, n = 798; and MCI only, n = 425. AD = Alzheimer's disease; CN = cognitively normal; EC = entorhinal cortex; HbA1c = glycated hemoglobin; LDL-C = low-density lipoprotein cholesterol; MCI = mild cognitive impairment.

**Supplementary Figure S2. Distribution of standardized cerebral amyloid burden in the primary analytic cohort.**

**Note:** The histogram shows the observed distribution of cerebral amyloid-beta burden expressed as a z-score among the 1,253 participants included in the primary complete-case analysis. The solid curve represents a kernel-density estimate, and rug marks along the x-axis indicate individual observations. The distribution is presented to show the empirical support across the predictor range used for the Aβ × LDL-C interaction analysis.

**Supplementary Figure S3. Distribution of LDL-C in the primary analytic cohort.**

**Note:** The histogram shows the observed distribution of circulating low-density lipoprotein cholesterol (LDL-C), expressed in mg/dL, among the 1,253 participants in the primary complete-case analysis. The solid curve represents a kernel-density estimate, and rug marks indicate individual observations. The 16th, 50th, and 84th percentiles used to probe the primary interaction corresponded to approximately 66, 100, and 138 mg/dL, respectively. These probe values were selected to characterize conditional statistical associations across the observed LDL-C distribution and should not be interpreted as clinical cutoffs.

**Supplementary Figure S4. Johnson–Neyman analysis using conventional ordinary least-squares covariance estimates.**

**Note:** The solid curve represents the estimated conditional association between standardized cerebral amyloid-beta (Aβ) burden and bilateral entorhinal cortex (EC) thickness across the continuous LDL-C distribution. The shaded band represents the conventional ordinary least-squares 95% confidence interval, and the horizontal dashed line denotes a conditional association of zero. Under conventional covariance estimation, the within-range Johnson–Neyman boundary occurred at LDL-C z = −0.1799, corresponding to approximately 96.5 mg/dL; approximately 52.8% of participants had LDL-C values above this boundary. A second mathematical boundary occurred at z = −2.2361 (approximately 23.9 mg/dL) but lay outside the observed LDL-C range. This figure is provided as a conventional-inference sensitivity comparison with the HC3 Johnson–Neyman analysis presented as Main Figure 2. Johnson–Neyman values are sample-specific statistical regions of significance and should not be interpreted as clinical thresholds.

**Supplementary Figure S5. Cook's distance diagnostic for the primary Aβ × LDL-C mediator model.**

**Note:** Cook's distance is shown for each of the 1,253 observations in the primary diagnosis-free Model 7 EC-thickness regression. The horizontal dashed line indicates the prespecified screening threshold of 4/N (approximately 0.00319). Open highlighted symbols identify observations exceeding this threshold. Seventy-three observations exceeded the Cook's distance screening criterion. Cook's distance was used as an influence diagnostic rather than as an automatic exclusion criterion; all participants were retained in the primary analysis. Influence sensitivity was further evaluated using interaction-specific DFBETAS, leave-one-participant-out analyses, and a conservative deletion stress test reported in Supplementary Table S5.

**Supplementary Figure S6. Comparison of Aβ × lipid interaction estimates across circulating lipid measures.**

**Note:** Points represent interaction coefficients from parallel diagnosis-free first-stage moderation models evaluating LDL-C, HDL-C, triglycerides, total cholesterol, and non-HDL cholesterol as moderators of the association between cerebral amyloid-beta (Aβ) burden and bilateral entorhinal cortex thickness. All analyses were performed in the identical lipid-complete sample (N = 1,251), and lipid variables were z-standardized before model fitting. Horizontal error bars represent HC3 heteroskedasticity-consistent 95% confidence intervals. Filled symbols indicate interactions that remained supported after Benjamini–Hochberg false-discovery-rate correction (q < 0.05), whereas open symbols indicate q ≥ 0.05. LDL-C and non-HDL cholesterol remained supported after HC3 inference and multiplicity correction, whereas HDL-C, triglycerides, and total cholesterol did not. These findings indicate that the moderation pattern should not be interpreted as uniquely specific to LDL-C and may instead reflect a broader atherogenic-cholesterol context.
